# Seasonality and Meteorological Factors Associated With Different Hand, Foot, and Mouth Disease: Serotype-Specific Analysis From 2010 to 2018 in Zhejiang Province, China

**DOI:** 10.3389/fmicb.2022.901508

**Published:** 2022-05-20

**Authors:** Yijuan Chen, Wanwan Sun, Feng Ling, Jimin Sun, Yanli Cao, Zhiping Chen, Ziping Miao

**Affiliations:** Department of Communicable Diseases Control and Prevention, Zhejiang Provincial Centers for Disease Control and Prevention, Hangzhou, China

**Keywords:** HFMD prediction, serotype, Zhejiang province, meteorological factors, seasonality

## Abstract

**Background:**

Hand-foot-mouth disease (HFMD) is caused by a group of enteroviruses (EVs) and has a high incidence in children; some subtypes had high mortalities in children. The subtypes of HFMD had a different incidence across seasons. Thereby, we suspect that the infection of HFMD is varied by meteorological factors. However, studies examining serotype-specific associations between meteorological factors and HFMD incidence were rare.

**Methods:**

We obtained all HFMD cases that occurred from 1 January 2010 to 31 December 2018 in Zhejiang province from the China Information System for Disease Control and Prevention (CISDCP). Daily meteorological data for Zhejiang province were provided by the China Methodological Data Sharing Service System and linked to HFMD cases based on residential addresses and dates of onset. The associations between meteorological factors and HFMDs were examined using distributed lag non-linear models (DLNMs) for each serotype.

**Results:**

Overall, the incidences of all HFMD cases were increasing in study years, while the number of severe and fatality cases were decreasing. The dominant serotypes varied by study year. The association between temperature and incidence of both CVA16 and EV71 serotypes showed an inverted U shape. The risk ratio for CVA16 was increasing when temperature is 11–25°C, reaching the maximum RR at 18°C and humidity above 77% can promote the occurrence with CVA16, and temperature between 11 and 32°C with the maximum RR at 21°C and relative humidity above 77% are risk conditions of the occurrence of HFMD associated with EV71. For other enteroviruses causing HFMD, temperature above 11°C and humidity above 76% have a risk effect. CVA16, EV71, and all enteroviruses of HFMD have a maximum effect on lag day 0, and temperature is 35, 34, and 33°C respectively, while the enteroviruses of HFMD other than EV71 and CVA16 has a maximum effect when the temperature is 33°C and the lag time is 7 days.

**Conclusion:**

This study shows that meteorological factors have an effect on the occurrence of different HFMD serotypes. Local control strategies for public health should be taken in time to prevent and reduce the risk of HFMD while the weather is getting warmer and wetter.

## Introduction

Hand-foot-mouth disease (HFMD) is an infectious disease that predominantly affects young children, especially those under 5 years old. HFMD can be caused by infection of several types of viruses such as Enterovirus 71 (EV71), Coxsackie virus A16 (CVA16), and other gastrointestinal viruses. Since May 2008, HFMD has been added into China's nationwide infectious disease surveillance and classified as a “Class C” infectious disease because of high incidence and fatalities of several subtypes. That is, any new case of HFMD is required to be reported to the surveillance system managed by China Center for Disease Control and Prevention (CDC). Until now, the total number of HFMD cases in China is around 2 million per year.

Majority of HFMD cases had mild symptoms and self-recovered in less than 2 weeks. However, the HFMD cases caused by specific types of virus (e.g., EV71) may result in severe complications affecting the central nervous and cardiopulmonary systems (Xu et al., 2012). Cases and deaths caused by a specific HFMD-related virus have resulted in great economic loss and social upheaval. For example, an outbreak of HFMD mainly caused by EV71 in the northern part of Anhui province in 2008 resulted in severe pediatric pneumonia pandemic (Zhang et al., 2010). Therefore, understanding the serotype-related risk factors for HFMD can aid in disease prevention and reduce the impact on public health.

Several bodies of evidence suggested a possible association between weather and the incidence of HFMD. First of all, we observed a seasonality of HFMD during our surveillance (Zi-Ping et al., 2012). Second, previous studies have reported that the incidence of HFMD was significantly influenced by meteorological factors, such as temperature, precipitation, and relative humidity (Ma et al., 2010; Zhao et al., 2018; Yu et al., 2019). Nevertheless, there is a research gap, because although the seasonality of HFMD is varied by the type of virus (Xie et al., 2020), research studies on the association between different HFMD serotypes and meteorology are very limited.

In this study, we aimed to investigate the association between multiple weather characteristics and the incidence of HFMD in Zhejiang province of China. Additionally, since we have obtained laboratory-confirmed results for a proportion of HFMD cases, we were able to examine the associations of interests by each type of HFMD virus. We hypothesized that meteorological conditions play a role in the incidence of HFMD, but that their importance was varied by different viruses. We expected to provide reference for the prevention of specific types of HFMD in order to reduce the risks of outbreaks.

## Methods

### Study Site

**Figure 1:**
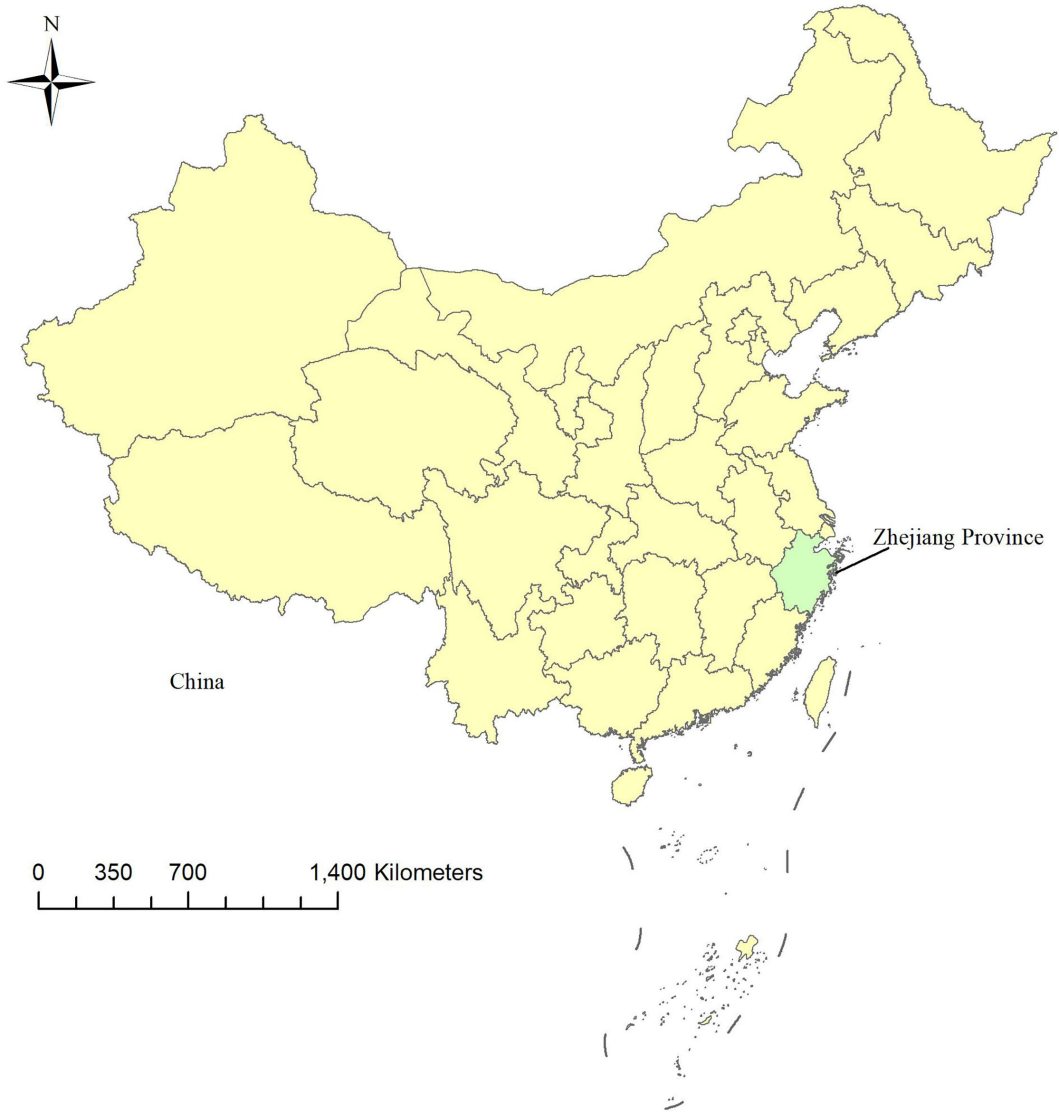
Location of Zhejiang province in China.

Zhejiang is a province located in the southeastern coast of China and had a population of ~56.7 million people in 2018. It lies between latitudes 27.05°N and 31.19°N and longitudes 118.03°E and 122.95°E. It is divided into 11 cites, which are further subdivided into 90 administrative units. It has four distinct seasons and a subtropical monsoon climate (Figure 1).

### Data Sources

HFMD cases in Zhejiang Province from 1 January 2010 to 31 December 2018 were extracted from the China Information System for Disease Control and Prevention (CISDCP), where all HFMD cases in China were required to report within 24 h of diagnosis. For the purpose of etiological monitoring, CDCs in all counties of Zhejiang province were required to collect 40 blood samples every month for laboratory testing. We obtained comprehensive data from CISDCP, including HFMD cases' demographic characteristics, clinic medical records, laboratory testing results, and residential addresses.

Daily meteorological data for the Zhejiang province were provided by the China Methodological Data Sharing Service System (https://data.cma.cn/). The meteorological variables include daily temperature, relative humidity, rainfall, air pressure, wind velocity, and sunshine hours. We matched the residential address of each case to the nearest meteorological station using ArcGIS Pro, and the meteorological and HFMD data were linked.

### Statistical Analysis

A descriptive analysis was conducted to examine the characteristics of HFMD cases and meteorological variables. The mean value and standard deviation were calculated for continuous variables. Percentage heatmaps were drawn to represent the change in the composition ratio of serotype classification of HFMD, and each grid represented the proportion of the number of cases of a genotype in the total number of cases of HFMD for that month and year.

The association between meteorological variables and the daily count of HFMD cases was examined based on a distributed lag non-linear model (DLNM). Daily counts of HFMD cases were assumed to have a Poisson distribution to account for over-dispersion. Daily average temperature, average relative humidity, and cumulative rainfall were incorporated in the model in the form of a “cross-basis” to account for a possible complex association among temperature, relative humidity, and HFMD incidence. Each model contains two elements: the cross-basis of the non-linear exposure-response association and the natural cubic spline of the covariant variables.

To analyze the lag-response effect of meteorological factors, temperature and relative humidity were applied to the cross-basis function of DLMN. When one factor was included in the function, the other one was set as a covariate variable in ns() function. Given that the longest incubation period of HFMD is approximately 14 days, we set the longest lag phase to 14.

(1) $$\begin{array}{r} \begin{array}{c} \log\left[E\left(Yt\right)\right]=\beta +\overset{14}{\underset{p=0}{\sum }}\alpha \;Temp_{t-p}+ns\;\left(RH_{t}\right)\\ +ns\left(Prep_{t}\right)+ns\left(Holiday_{t}\right). \end{array} \end{array}$$

Here, (*Yt*) means the estimated daily count of HFMD on day *t*, *E*(*Yt*) denotes the expectation of *Yt*. β stands for the intercept of the whole equation. *Temp*_*t*−*p*_ is a matrix obtained by DLNM to model non-linear and distributed lag effects of temperature over the *t* day to lag *p* days, and α is the vector of coefficients for *Temp*_*t*−*p*_. ns() represents the natural spline. The relative risk (RR) and 95% confidence interval (CI) of HFMD associated with each 1-degree of 1-percent increases in temperature or relative humidity were calculated. The optimal degrees of freedom (df) for the spline function were estimated by generalized cross-validation (GCV) criteria.

All the analyses in our study were performed using the “dlnm,” “mgcv,” and “pheatmap” packages in the R software (version 3.6.1). The CI of all two-sided statistical tests in the study was set to 95%, and *P* < 0.05 was considered statistically significant.

## Results

### Prevalence and Etiological Characteristics of HFMD in Zhejiang Province

**Figure 2:**
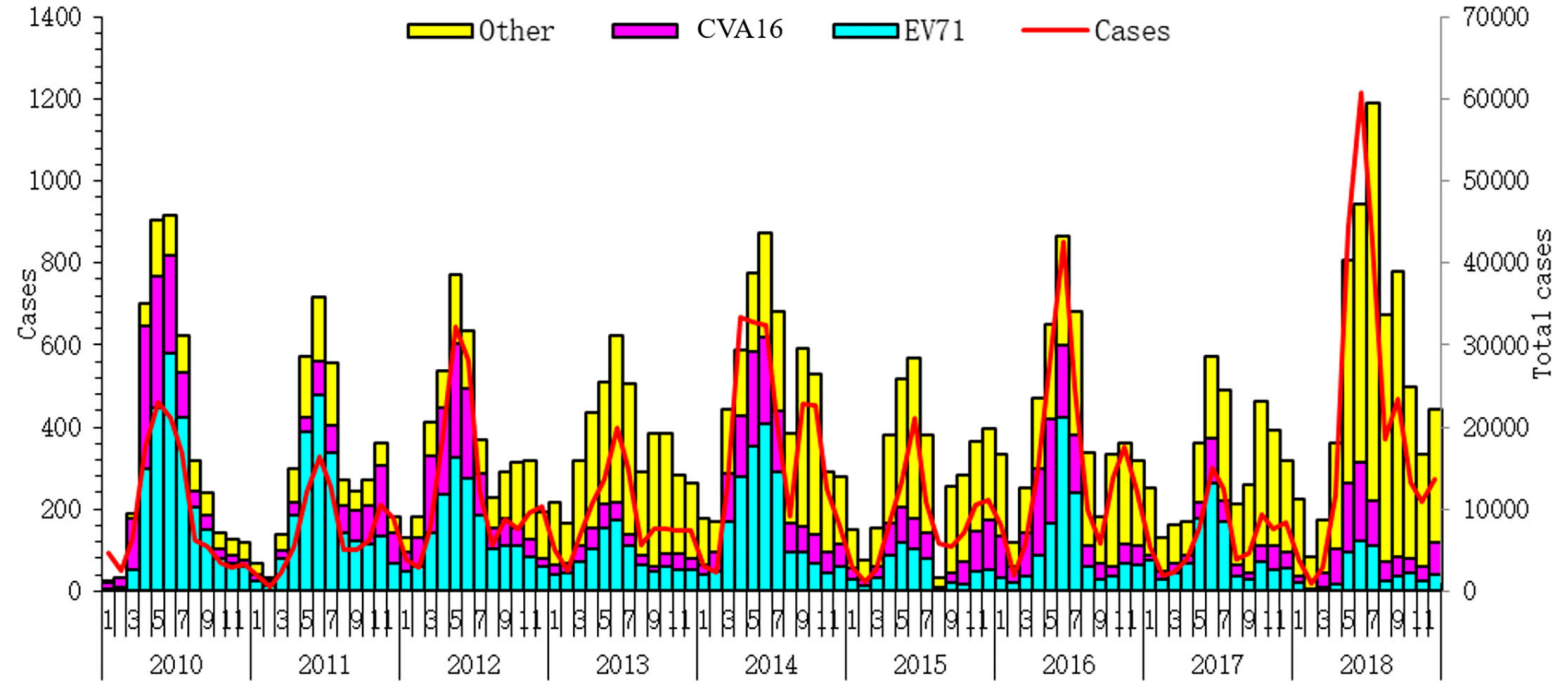
Total number of hand-foot-mouth disease (HFMD) cases and serotype-specific cases from 2010 to 2018 in Zhejiang province.

From 2010 to 2018, a total of 1,284,821 HFMD cases in Zhejiang were reported to China's nationwide infectious disease surveillance. Among them, 42,104 cases were laboratory-confirmed, including 12,785 (30.37%) EV71 cases, 8,517 (20.23%) CVA16 cases, and 20,802 (49.41%) other enterovirus cases that were reported in the Zhejiang province (Supplementary Table 1; Figure 2).

### Time Trends and Seasonality Effects

The weekly summary of HFMD cases and linked meteorological factors are presented in Table 1. The average number of HFMD cases was 88.27 (range from 0 to 423) per week. During the study period, the mean weekly values of temperature, humidity, and cumulative precipitation were 17.51°C, 75.25%, and 27.67 mm, respectively (Table 1).

**Table 1:** Weekly summary of hand-foot-mouth disease (HFMD) cases and meteorological factors in Zhejiang province, China, 2010–2018.

**Variable**	**Mean**	**S.D**.	**Min**	**25%**	**50%**	**75%**	**Max**
Number of cases	88.27	62.59	0	44	78	121.5	423
Temperature (°C)	17.51	8.43	0.53	9.94	18.00	24.66	32.75
Relative humidity (%)	75.25	8.38	31.89	70.51	76.01	80.90	93.54
Precipitation (mm)	29.67	31.71	0.00	7.41	21.47	39.40	251.52

*SD, standard deviation; Min, minimum; Max, maximum*.

**Figure 3:**
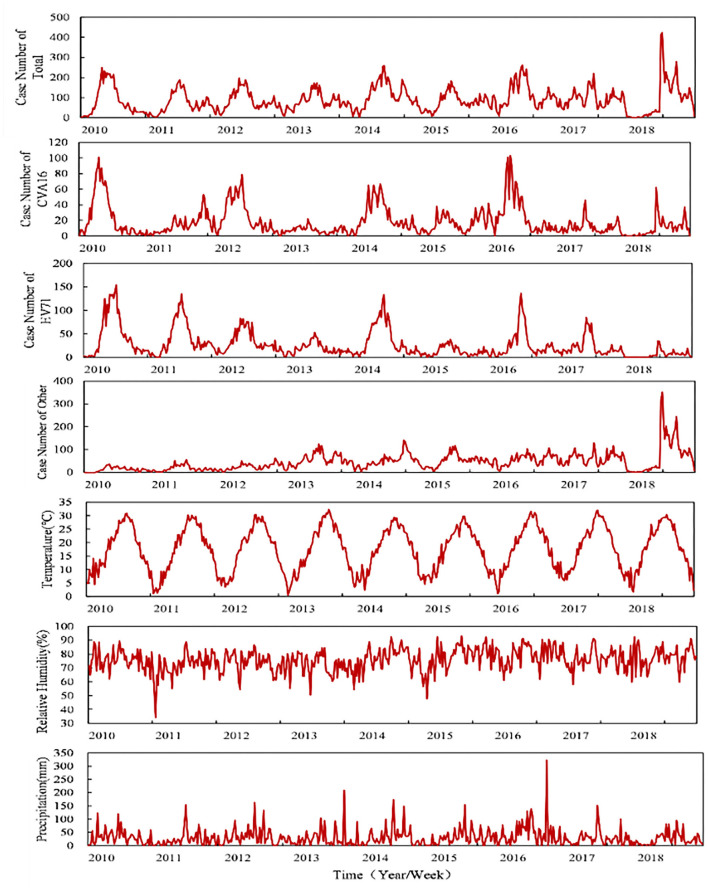
Distributions of the three categories of HFMD and meteorological factors from 2010 to 2018.

Figure 3 displays the time series distribution of the weekly number of HFMD cases of total and three categories of CVA16, EV71, and other enteroviruses causing HFMD, weekly average temperature, weekly average relative humidity, and weekly total precipitation during our study period in Zhejiang, China. The long-term trend of the number of HFMD cases was seasonality, with peaking in spring (the 15–30 weeks of the year).

**Figure 4:**
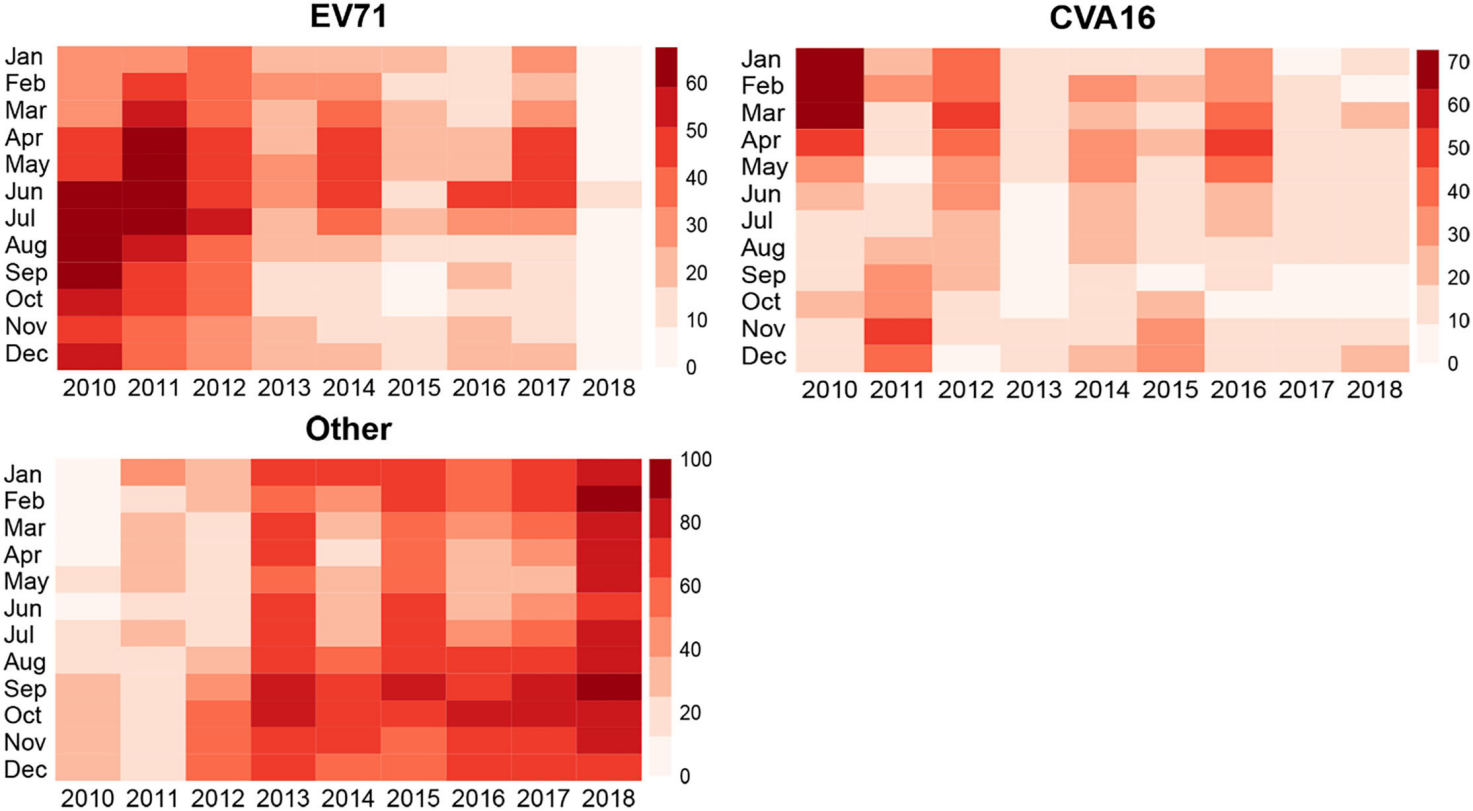
Relationships between different enteroviruses of HFMD cases and temperature and humidity in Zhejiang province, China from 2010 to 2018.

Figure 4 shows t the proportion (%) of each serotype among all laboratory-confirmed cases. The leading serotype varied between seasons and between our study years. Specifically, EV71 was the leading serotype in late spring to early fall in the first 2 years of our study (2010–2011). CVA16 was the leading serotype for the winter of 2010. However, the cases of both serotypes were decreased in recent study years. Since 2013, other enteroviruses have been predominantly leading the cases of HFMD in Zhejiang.

### Temperature, Relative Humidity, and EV71 Infection Activity

**Figure 5:**
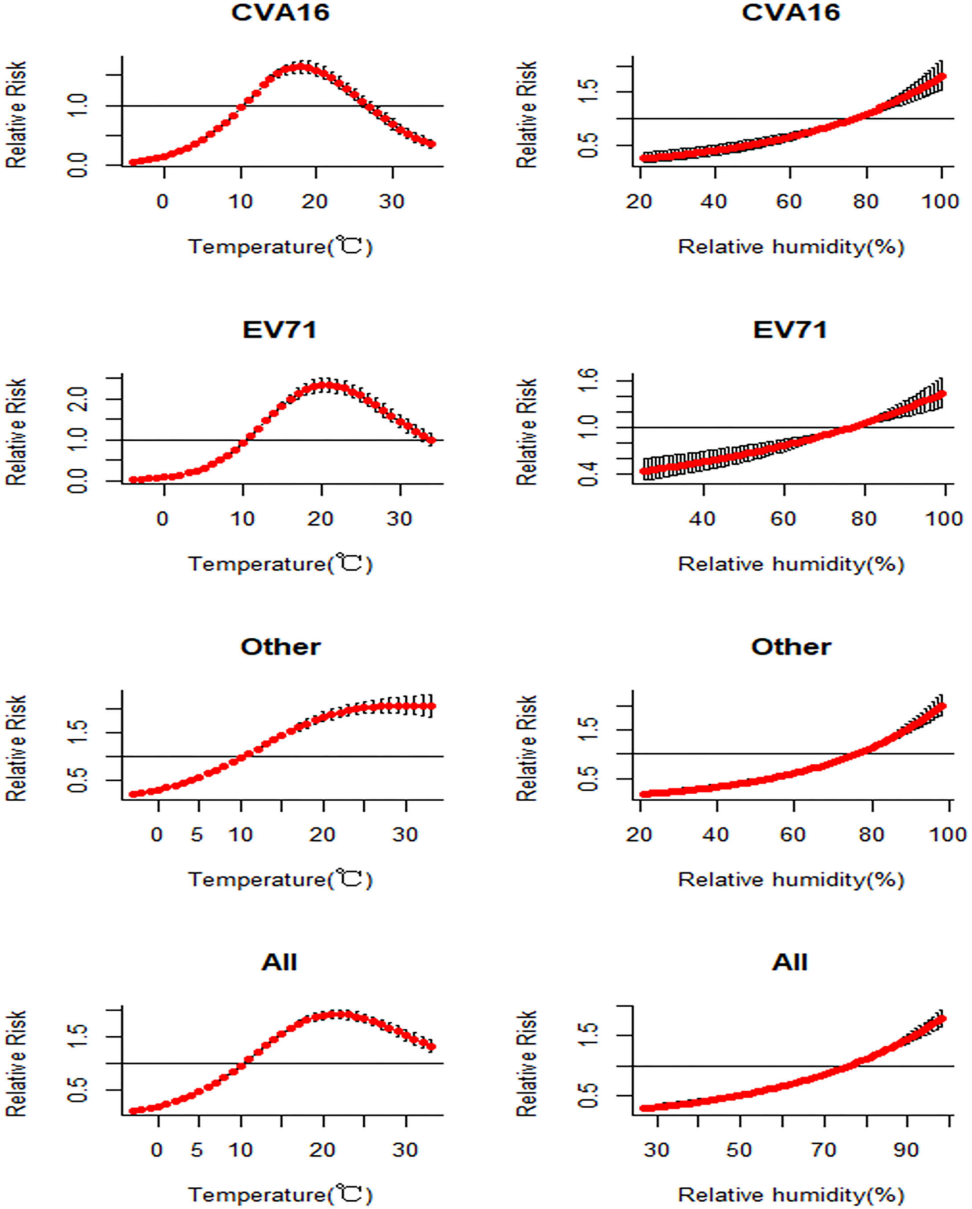
Associations between incidence of HFMD and meteorological factors estimated using a case-crossover design by serotype.

Figure 5 shows the associations between serotype-specific HFMD and meteorological factors (i.e., temperature or humidity) estimated using a case-crossover design. In our results, the all serotypes (i.e., EV71, CVA16, and other enteroviruses of HFMD) have a significant association with meteorological factors. The association between temperature and CVA16 shows an inverted U shape. That is, when temperature ranged between 11 and 25°C (${\text{RR}}_{25{}^{\circ}\text{C}}$ = 1.169; 95% CI: 1.076–1.271), temperature is positively associated with the occurrence of HFMD, and its effect reaches maximum at 18°C (${\text{RR}}_{18{}^{\circ}\text{C}}$ = 1.642; 95% CI: 1.542–1.749). When temperature is higher than 28°C (${\text{RR}}_{28{}^{\circ}\text{C}}$ = 0.864; 95% CI: 0.776–0.962), the association becomes negative. Humidity higher than 77% (RR_77%_ = 1.013; 95% CI: 1.009–1.016) was a condition of promoting the occurrence of HFMD.

Similarly, when temperature is between 11 and 32°C, its association with the occurrence of EV71 is positive (${\text{RR}}_{32{}^{\circ}\text{C}}$ = 1.213; 95% CI: 1.065–1.382), with the maximum effect at 21°C (${\text{RR}}_{21{}^{\circ}\text{C}}$ = 2.335; 95% CI: 2.169–2.513). Relative humidity higher than 77% (RR_77%_ = 1.01; 95% CI: 1.006–1.013) represents the risk condition of the occurrence of HFMD.

There was a positive correlation between the other enteroviruses and the two meteorological factors (temperature and humidity). However, the associations are inversed when temperature reaches 11°C and humidity reaches 76%. Before that, the increase in temperature or humidity is associated with lower risk of HFMD cases caused by other enteroviruses. When temperature or humidity becomes higher, the association becomes positive (${\text{RR}}_{11{}^{\circ}\text{C}}$ = 1.056; 95% CI: 1.049–1.063), and humidity higher than 76% (RR_76%_ = 1; 95% CI: 1) has a risk effect with other enteroviruses of HFMD and can promote the occurrence of HFMD related with other enteroviruses of HFMD.

All enteroviruses of HFMD have a significant risk effect when temperature value was between 11 and 33°C (${\text{RR}}_{33{}^{\circ}\text{C}}$ = 1.307; 95% CI: 1.197–1.427). When temperature reaches 22°C, the effect of temperature on HFMD reaches maximum (${\text{RR}}_{22{}^{\circ}\text{C}}$ = 1.91; 95% CI: 1.83–1.993). Humidity higher than 77% (RR_77%_ = 1.022; 95%CI: 1.019–1.025) has a risk effect on HFMD on the whole.

**Figure 6:**
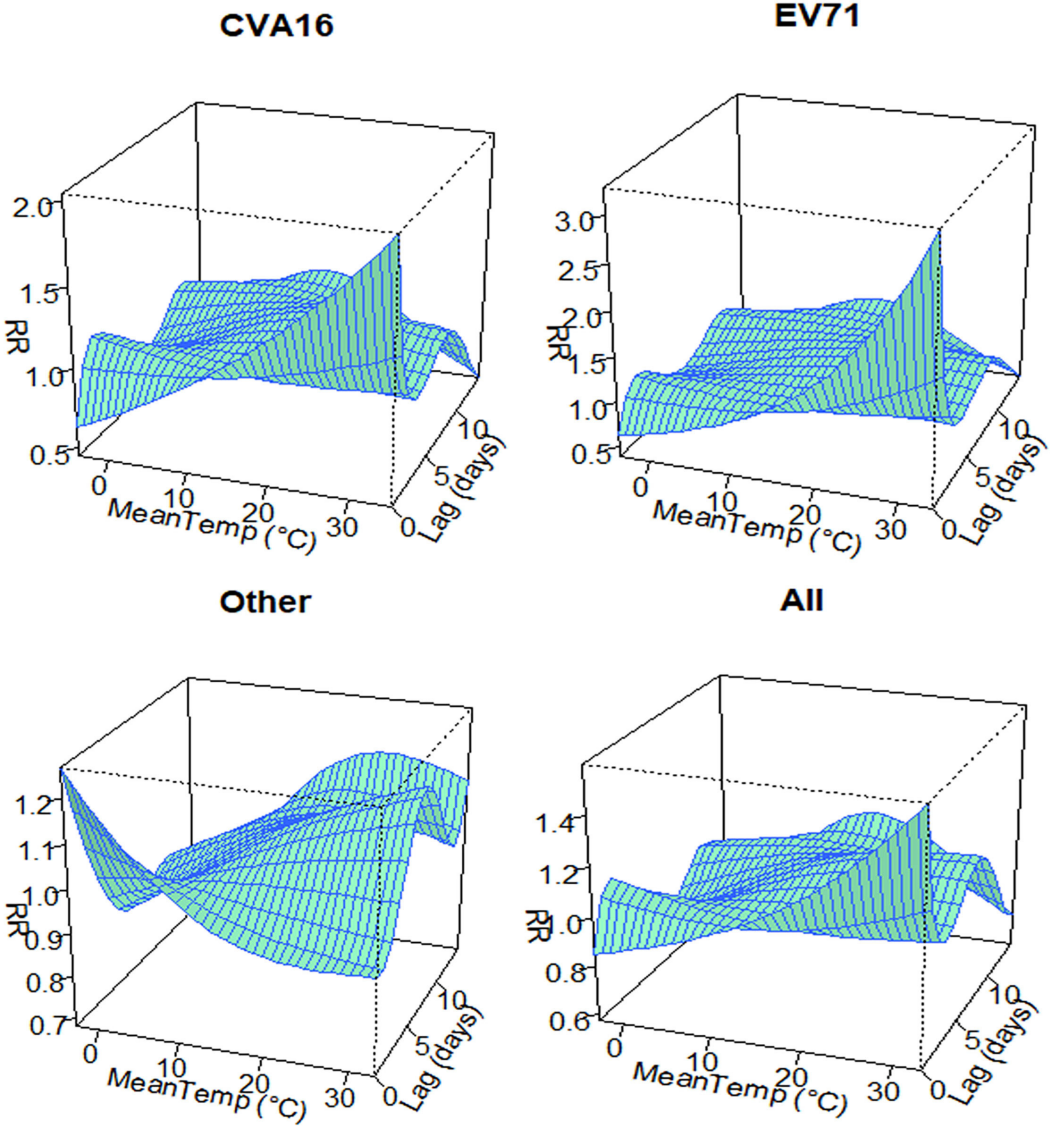
Relative risks of daily HFMD by daily mean temperature along 14 lag days in Zhejiang province, China from 2010 to 2018.

The three-dimensional plot of the associations between serotype-specific HFMD cases and temperatures at lag 0–lag 14 days is shown in Figure 6. When the lag time of CVA16 is 0 and the temperature is 35°C, the RR value of CVA16 reaches its maximum (2.047; 95% CI: 1.437–2.916). Similarly, for EV71, the RR value reaches its maximum (3.286; 95% CI: 2.475–4.361) when temperature is 34°C (lag = 0). The maximum of RR (1.196; 95% CI: 1.094–1.308) for other serotypes of HFMD was observed at 33°C and lag = 7. The maximum of RR (1.596; 95% CI: 1.335–1.909) for all the HFMD cases was observed at 33°C and lag = 0.

## Discussion

HFMD remains the most common infectious disease among infants and young children in almost all provinces of China. From 2008 to 2015, ~13.8 million HFMD cases were reported in mainland China, including 130,000 serious cases and 3,300 deaths, while during the same period, a total of 875,945 HFMD cases ranking among the top five of notifiable diseases were identified in Zhejiang province (Chen et al., 2016).

In this study, we characterized HFMD cases using the data extracted from the national surveillance for 10 years (2008–2018). Overall, the incidence of HFMD in Zhejiang province was increasing during our study years, but the numbers of severe cases and mortality were both decreasing. This pattern was consistent to those reported in Zhang (2019). It should be noted that over the study years, the leading serotypes shifted from time to time. In the early years of this study, EV71 was the dominant serotype for all-type HFMD incidence and severe or fatal cases. Also, CVA16 had a relatively high incidence and was a major reason of HFMD-related herpetic pharyngitis. However, the composition of HFMD serotypes has changed since 2013. Serotypes and variants related to the epidemic cycles appeared more diverse, especially Coxsackie virus. The virus group A type 6 (CV-A6) and type 10 (CV-A10) brought different epidemic trends and atypical clinical manifestations, and the composition of other enteroviruses with severe outcomes increased gradually from 2009 to 2011 (Lu et al., 2012).

The associations between meteorological factors (e.g., temperatures, relative humidity, wind velocity, sunshine duration, and precipitation) and HFMD have been investigated in different Chinese cities by previous studies (Tian et al., 2018; Fu et al., 2019; Yan et al., 2019; Yu et al., 2019; Wang and Li, 2020). Most of the studies have focused on total HFMD cases. However for different predominant viruses, the suitable temperature conditions for growth, multiplication, and spread are not alike, nor is their tolerance for thermal effect (Gopalkrishna et al., 2012). We found that only one study in Taiwan, China compared the relationship between one pathogen of HFMD and meteorological factors, and reported that the rate of EV71 infection increased significantly with increase in daily temperature and relative humidity (Chang et al., 2012). According to our knowledge, this is the first study to investigate the association between meteorological factors and HFMD risk by different serotypes.

Most of the above-mentioned studies reported that the association between ambient temperature and total HFMD incidence showed an inverted U shape. Our study found that the association between temperature and CVA16 and between temperature and EV71 also showed a similar inverted U shape. The corresponding temperature of the maximum RR was higher for EV71 than CVA16, indicating that EV71 can tolerate higher ambient temperature than CVA16, therefore outbreaks of EV71 are more likely to occur in the summer-time. For other serotypes of HFMD, they showed a consistent positive association with temperature when it was above 11°C. The associations between humidity and all the studied HFMD serotypes had a similar linear trend, indicating an increased risk of HFMD when humidity was above 75%. It is important to point out that the types of non-EV71 and non-CVA16 enteroviruses in our study mainly refer to CVA6 and CVA10, which account for most of the other enteroviruses according to the literature (Lu et al., 2012; Xie et al., 2020).

Several limitations merit consideration. In China's nationwide surveillance system, HFMD cases are reported by healthcare providers. Thus, only patients who visited a clinic or received treatment were included. Therefore, the number of HFMD cases in this study may be underestimated. In addition, while all samples were examined PCR by pan-enterovirus detection kit which is a preliminary screening test, we only have the laboratory-confirmed data for EV71, CVA16, and others without further identification, and classification of other serotypes into the same group might lead to bias for the composition of pathogens various in different years. Future studies with more detailed serotypes are recommended.

## Data Availability Statement

The datasets presented in this article are not readily available because the dataset was extracted from the China Information System for Disease Control and Prevention (CISDCP). Requests to access the datasets should be directed to yjchen@cdc.zj.cn.

## Author Contributions

YC: data curation, conception, software, and writing (original draft). WS: methodology and software. ZC, FL, and JS: supervision. YLC: data collection. YC and ZM: conception, supervision, and writing (review). All authors reviewed the manuscript.

## Funding

This study was supported by the Key Research and Development Project of Zhejiang Province, China (Grant No: 2018C03070).

## Conflict of Interest

The authors declare that the research was conducted in the absence of any commercial or financial relationships that could be construed as a potential conflict of interest.

## Publisher's Note

All claims expressed in this article are solely those of the authors and do not necessarily represent those of their affiliated organizations, or those of the publisher, the editors and the reviewers. Any product that may be evaluated in this article, or claim that may be made by its manufacturer, is not guaranteed or endorsed by the publisher.
