# Supplementary material for: Seasonality and Meteorological Factors Associated With Different Hand, Foot, and Mouth Disease: Serotype-Specific Analysis From 2010 to 2018 in Zhejiang Province, China
Source: Front Microbiol. 2022 May 20;13:901508. doi: 10.3389/fmicb.2022.901508 (PMC9164151; doi:10.3389/fmicb.2022.901508)
Supplement: Supplementary file 1 [file Table_1.DOCX]

Supp. table 1 Number of HFMD cases from 2010 to 2018 in Zhejiang Province

| Year | Month | Number of identified  EV71 cases | Number of identified  CVA16 cases | Number of identified  other cases | Total  Cases |
| --- | --- | --- | --- | --- | --- |
| 2010 | 1 | 5 | 16 | 1 | 4598 |
|  | 2 | 8 | 25 | 0 | 2432 |
|  | 3 | 52 | 124 | 13 | 6406 |
|  | 4 | 300 | 347 | 56 | 17618 |
|  | 5 | 449 | 319 | 137 | 23119 |
|  | 6 | 580 | 240 | 95 | 21116 |
|  | 7 | 424 | 108 | 91 | 16848 |
|  | 8 | 203 | 42 | 74 | 6136 |
|  | 9 | 149 | 35 | 56 | 5397 |
|  | 10 | 78 | 24 | 42 | 3555 |
|  | 11 | 66 | 21 | 38 | 2982 |
|  | 12 | 59 | 15 | 46 | 3237 |
| 2011 | 1 | 25 | 15 | 29 | 1899 |
|  | 2 | 12 | 13 | 8 | 548 |
|  | 3 | 81 | 20 | 39 | 2395 |
|  | 4 | 187 | 31 | 79 | 5490 |
|  | 5 | 387 | 37 | 150 | 12076 |
|  | 6 | 477 | 84 | 157 | 16434 |
|  | 7 | 339 | 65 | 153 | 12581 |
|  | 8 | 143 | 64 | 63 | 5100 |
|  | 9 | 121 | 75 | 48 | 5001 |
|  | 10 | 114 | 95 | 61 | 6212 |
|  | 11 | 136 | 169 | 55 | 10476 |
|  | 12 | 67 | 75 | 40 | 8941 |
| 2012 | 1 | 47 | 50 | 35 | 3784 |
|  | 2 | 61 | 68 | 51 | 2850 |
|  | 3 | 144 | 186 | 81 | 7609 |
|  | 4 | 234 | 213 | 90 | 18964 |
|  | 5 | 324 | 278 | 169 | 32261 |
|  | 6 | 276 | 217 | 143 | 28122 |
|  | 7 | 187 | 101 | 81 | 12207 |
|  | 8 | 102 | 50 | 77 | 5669 |
|  | 9 | 111 | 68 | 113 | 8839 |
|  | 10 | 109 | 46 | 158 | 7649 |
|  | 11 | 82 | 44 | 192 | 9494 |
|  | 12 | 58 | 21 | 113 | 10393 |
| 2013 | 1 | 42 | 23 | 152 | 5072 |
|  | 2 | 43 | 24 | 99 | 2444 |
|  | 3 | 70 | 41 | 209 | 6398 |
|  | 4 | 103 | 50 | 284 | 10591 |
|  | 5 | 154 | 58 | 296 | 13642 |
|  | 6 | 175 | 41 | 406 | 19841 |
|  | 7 | 111 | 26 | 369 | 14776 |
|  | 8 | 64 | 23 | 203 | 5716 |
|  | 9 | 47 | 13 | 326 | 7671 |
|  | 10 | 60 | 31 | 293 | 7538 |
|  | 11 | 52 | 39 | 192 | 7359 |
|  | 12 | 54 | 27 | 183 | 7484 |
| 2014 | 1 | 40 | 25 | 113 | 3141 |
|  | 2 | 49 | 47 | 72 | 2221 |
|  | 3 | 171 | 116 | 158 | 13082 |
|  | 4 | 278 | 151 | 160 | 33378 |
|  | 5 | 354 | 231 | 190 | 32840 |
|  | 6 | 409 | 209 | 255 | 32347 |
|  | 7 | 291 | 150 | 241 | 20510 |
|  | 8 | 95 | 69 | 220 | 9157 |
|  | 9 | 94 | 63 | 435 | 22783 |
|  | 10 | 66 | 71 | 394 | 22572 |
|  | 11 | 46 | 48 | 198 | 12498 |
|  | 12 | 58 | 58 | 162 | 8007 |
| 2015 | 1 | 30 | 25 | 94 | 2847 |
|  | 2 | 12 | 15 | 47 | 1158 |
|  | 3 | 34 | 26 | 94 | 2657 |
|  | 4 | 88 | 77 | 216 | 8176 |
|  | 5 | 117 | 86 | 314 | 13537 |
|  | 6 | 102 | 75 | 393 | 21133 |
|  | 7 | 80 | 63 | 237 | 10889 |
|  | 8 | 2 | 8 | 24 | 5754 |
|  | 9 | 22 | 22 | 213 | 5376 |
|  | 10 | 17 | 53 | 214 | 7207 |
|  | 11 | 48 | 99 | 219 | 10447 |
|  | 12 | 53 | 121 | 221 | 11037 |
| 2016 | 1 | 31 | 105 | 197 | 8061 |
|  | 2 | 20 | 39 | 61 | 1969 |
|  | 3 | 35 | 107 | 109 | 5557 |
|  | 4 | 88 | 212 | 169 | 14790 |
|  | 5 | 165 | 253 | 231 | 28880 |
|  | 6 | 422 | 176 | 266 | 42567 |
|  | 7 | 241 | 139 | 302 | 24892 |
|  | 8 | 58 | 52 | 227 | 9959 |
|  | 9 | 30 | 38 | 114 | 5824 |
|  | 10 | 38 | 21 | 273 | 13770 |
|  | 11 | 67 | 47 | 248 | 17634 |
|  | 12 | 64 | 46 | 210 | 12119 |
| 2017 | 1 | 77 | 10 | 165 | 5355 |
|  | 2 | 27 | 21 | 83 | 1864 |
|  | 3 | 45 | 22 | 95 | 2342 |
|  | 4 | 66 | 21 | 83 | 3809 |
|  | 5 | 176 | 42 | 144 | 7733 |
|  | 6 | 263 | 109 | 200 | 15077 |
|  | 7 | 170 | 51 | 270 | 12436 |
|  | 8 | 38 | 26 | 148 | 3860 |
|  | 9 | 27 | 18 | 215 | 4676 |
|  | 10 | 73 | 38 | 352 | 9318 |
|  | 11 | 54 | 57 | 282 | 7649 |
|  | 12 | 55 | 41 | 224 | 8297 |
| 2018 | 1 | 22 | 15 | 187 | 3848 |
|  | 2 | 1 | 6 | 77 | 933 |
|  | 3 | 10 | 33 | 129 | 2842 |
|  | 4 | 17 | 85 | 260 | 11743 |
|  | 5 | 96 | 169 | 542 | 44279 |
|  | 6 | 122 | 192 | 631 | 60714 |
|  | 7 | 109 | 113 | 970 | 42627 |
|  | 8 | 23 | 50 | 599 | 18453 |
|  | 9 | 38 | 47 | 696 | 23347 |
|  | 10 | 43 | 37 | 418 | 13232 |
|  | 11 | 23 | 38 | 273 | 10972 |
|  | 12 | 41 | 76 | 326 | 13669 |
